# Prevalence and management of dysphagia in nursing home residents in Europe and Israel: the SHELTER Project

**DOI:** 10.1186/s12877-022-03402-y

**Published:** 2022-08-31

**Authors:** Giuseppina Dell’Aquila, Nikolina Jukic Peladic, Vanessa Nunziata, Massimiliano Fedecostante, Fabio Salvi, Barbara Carrieri, Rosa Liperoti, Angelo Carfì, Paolo Eusebi, Graziano Onder, Paolo Orlandoni, Antonio Cherubini

**Affiliations:** 1 Geriatria, Accettazione geriatrica e Centro Di Ricerca Per L’invecchiamento, IRCCS INRCA, Via della Montagnola 81, 60127 Ancona, Italy; 2 Clinical Nutrition Unit, IRCCS INRCA Ancona, Via della Montagnola 81, 60127 Ancona, Italy; 3 Geriatrics and Geriatric Emergency Care, Italian National Research Center On Aging (IRCCS-INRCA), Via della Montagnola, 81, 60127 Ancona, Italy; 4 Fondazione Policlinico Universitario A. Gemelli IRCCS and Università Cattolica del Sacro Cuore, Rome, Italy; 5 Department of Cardiovascular, Endocrine-Metabolic Diseases and Aging, Istituto Superiore Di Sanità, Via Giano della Bella 34, 00161 Rome, Italy

**Keywords:** Nursing home, Dysphagia, Artificial nutrition, Texture modified diet, Loss of weight, Mortality

## Abstract

**Background:**

Dysphagia is a frequent condition in older nursing home residents (NHRs) which may cause malnutrition and death. Nevertheless, its prevalence is still underestimated and there is still debate about the appropriateness and efficacy of artificial nutrition (AN) in subjects with severe dysphagia. The aim is to assess the prevalence of dysphagia in European and Israeli NHRs, its association with mortality, and the relationship of different nutritional interventions, i.e. texture modified diets and AN—with weight loss and mortality.

**Methods:**

A prospective observational study of 3451 European and Israeli NHRs older than 65 years, participating in the SHELTER study from 2009 to 2011, at baseline and after 12 months. All residents underwent a standardized comprehensive evaluation using the interRAI Long Term Care Facility (LTCF). Cognitive status was assessed using the Cognitive Performance Scale (CPS), functional status using Activities of Daily Living (ADL) Hierarchy scale. Trained staff assessed dysphagia at baseline by clinical observation. Data on weight loss were collected for all participants at baseline and after 12 months. Deaths were registered by NH staff.

**Results:**

The prevalence of dysphagia was 30.3%. During the one-year follow-up, the mortality rate in subjects with dysphagia was significantly higher compared with that of non-dysphagic subjects (31.3% vs 17.0%,*p* = 0,001). The multivariate analysis showed that NHRs with dysphagia had 58.0% higher risk of death within 1 year compared with non-dysphagic subjects (OR 1.58, 95% CI, 1.31–1.91). The majority of NHRs with dysphagia were prescribed texture modified diets (90.6%), while AN was used in less than 10% of subjects. No statistically significant difference was found concerning weight loss and mortality after 12 months following the two different nutritional treatments.

**Conclusions:**

Dysphagia is prevalent among NHRs and it is associated with increased mortality, independent of the nutritional intervention used. Noticeably, after 12 months of nutritional intervention, NHRs treated with AN had similar mortality and weight loss compared to those who were treated with texture modified diets, despite the clinical conditions of patients on AN were more compromised.

## Introduction

Age-related physiological changes of the swallowing mechanism, the so-called presbyphagia, predominantly consist of a slowing and weakening of the different stages of deglutition: oral, pharyngeal and esophageal [1]. These changes are not always indicative of an impairment and may even be functional to safe swallowing [2–7]. In some cases, though, age-related changes could contribute to the onset of dysphagia i.e. the difficulty or impossibility in forming or moving a bolus efficiently and safely from the oral cavity to the esophagus [8]. The most common type among older subjects is oropharyngeal dysphagia [9]. Although some studies have identified the age as a significant single risk factor for dysphagia, changes in swallowing resulting from ageing are more frequently co-responsible for it, along with some diseases and the medicines used to treat them [6, 10–12]. Dysphagia is closely related to neurological and neurodegenerative diseases such as stroke, dementia and Parkinson’s disease, but also to sarcopenia, a skeletal muscle disorder due to loss of strength and muscle mass. [13–16]**.** Medication related adverse events, i.e. xerostomia, cognitive impairment and reduction of alertness, as well as polypharmacy, i.e. taking five or more medications daily, are also important risk factors for dysphagia [17–20]. In its initial stages, dysphagia may cause distress and anxiety, negatively affecting the quality of life of affected subjects [21]. Over time it may cause many life-threatening clinical conditions, such as undernutrition and dehydration, respiratory infections, aspiration pneumonia, and even death [22–24]**.** Nevertheless, despite the progressive aging of the population has made dysphagia an important concern for the public health, the knowledge on its prevalence and on the most appropriate methods of treatment presents still important knowledge gaps [25].

Studies have been performed using different screening and assessment tools, often including only small samples [26–28], thus making it difficult to define its prevalence, although it is known that dysphagia is particularly common among NHRs who have several risk factors: old age, neurological diseases, multimorbidity and polypharmacy [29–32].

Moreover, despite the evidence supporting the effectiveness of texture modified diets is still incomplete, the management of dysphagia in older subjects mainly consists in the adoption of that compensatory strategy whose aim is to prevent the onset of complications [33–36]. In the case of severe dysphagia, (AN)—enteral (EN) or parenteral (PN) -, is often prescribed [37]. However, the use of AN in severe dysphagia is still controversial and its effectiveness is often disputed.

We analyzed data from a large sample of NHRs who participated in the SHELTER study—the Services and Health for Elderly in Long TERm Care -, in order to investigate the prevalence of dysphagia in NHs, its association with mortality, and the effect of different types of nutritional intervention on weight loss and mortality.

## Methods

### SHELTER study sample

The SHELTER study is a prospective multinational cohort study that was performed from 2009 to 2011 in 57 Nursing Homes (NHs) of 7 European Union countries (Czech Republic, England, Finland, France, Germany, Italy, and The Netherlands) and 1 non-EU country (Israel). The detailed methodology of the study has been previously described [38]. Older adults residing in participating NHs at the beginning of the study and those admitted in the 3-month enrolment period following the initiation of the study were invited by each NH to participate in the study. Ethical approval was obtained from the ethics committees of the participating centers and informed consent was obtained from all subjects or their legal guardian. Subjects who accepted to participate and signed the written consent were assessed at baseline and reassessed after 12 months. The study was in Accordance with the Declaration of Helsinki. All experiments were performed in accordance with relevant guidelines and regulations.

### Data collection

#### Independent variables

Trained staff of each NH collected the data. At baseline, and after 12 months subjects underwent a standardized comprehensive evaluation using a validated instrument, i.e. the interRAI LTCF translated from the original English version into the languages of participating countries [39]. Multi-item summary scales embedded in the interRAI LTCF were used to measure residents’ characteristics. Cognitive status was assessed using the 7-point Multidimensional scaling (MDS) CPS, which combines information on memory impairment, level of consciousness, and executive function [40]. CPS score ranges from 0 to 6: a score ≥ 2 is diagnostic for dementia (comparable to MiniMental Status Examination (MMSE) ≤ 19), and a score higher than 4 indicates the presence of severe dementia. To evaluate functional status, the seven points MDS ADL Hierarchy scale, which groups activities of daily living according to the stage of the disablement process, was used [41]. The ADL Hierarchy scale ranges from 0 (no impairment) to 6 (total dependence). Medical history was obtained from the clinical records. Dysphagia was assessed by trained staff at baseline by clinical observation, without instrumental procedures. The evaluation included gathering information regarding the current swallowing problem, reviewing medical history, observing signs relevant to the residents’ medical status, observing the speech and swallowing structure, observing a patient during trial swallows, collecting data on nutrition therapy and diets and interviewing the personnel responsible for feeding assistance during meals. Subjects were grouped into two categories: dysphagic and non-dysphagic. Data on weight loss ≥ 5.0% in the last 3 months and/or ≥ 10.0% in the last 6 months were collected for all participants. Weight was registered after 12 months and the deaths have been recorded.

### Statistical analysis

Continuous variables were described with means and standard deviations, while categorical ones were reported as count and percentages. The data were analyzed to test for significant differences of clinical and demographic variables between patients with and without dysphagia. Student T test was used for continuous variables and chi-square test for categorical ones. Nonparametric alternatives were used whenever appropriate. Predictors of mortality in the whole population were assessed using multivariate Cox proportional hazards regression models. In the absence of a conversion event, data were censored at the most recent clinic visit. After having assessed the role of each risk factor, a *p*-value lower than 0.20 was used as screening criterion to consider the risk factor as candidate for the multivariate analysis. This decreased the probability of incorrect rejection of potentially important variables due to uncontrolled confounding. Backward elimination based on the Akaike’s information criterion was used to select a final model. NHs were considered as strata in the Cox regression. Hazard proportionality was assessed through analysis of scaled Schoenfeld residuals whereas martingale residuals were plotted against continuous covariates to detect nonlinearity. A regression logistic model was used to compare weight loss in subjects with dysphagia treated with artificial nutrition vs those treated with modified diet. Multivariate Cox proportional hazards regression model was used to compare mortality in subjects with dysphagia treated with artificial nutrition vs those treated with modified diet. Significance level of 5% was assumed for all the analyses. Statistical analyses were performed using R software version 4.1

## Results

A total of 3451 NHRs were enrolled in the SHELTER study and were assessed for dysphagia. Swallowing difficulties were registered in 30.3% of subjects. Residents with dysphagia were significantly older than non-dysphagic residents and the prevalence of multimorbidity and other risk factors for dysphagia—severe dementia Parkinson’s disease, cerebrovascular diseases—was also significantly higher in subjects with swallowing difficulties (see Table 1). The number of drugs was higher in non-dysphagic residents.

**Table 1:** Baseline sociodemographic, functional, and clinical parameters according to the presence or absence of dysphagia N (%) or mean ± SD

	**Total sample** **(*n* = 3451)**	**Dysphagia** **(*n* = 1046)**	**No- dysphagia** **(*n* = 2405)**
Age (years) mean±SD^a^	84.7 ± 7.7	85.4 ± 7.6	84.4 ± 7.7*
Sex (Female) n (%)^b^	2575 (74.6)	810 (77.4)	1765 (73.4)
Number of diseases mean±SD^a^	4.1 ± 2.7	4.5 ± 3	3.9 ± 2.6*
Number of drugs mean±SD^a^	7.0 ± 3.6	6.6 ± 3.4	7.2 ± 3.6*
ADL (0-6) mean±SD^a^	3.4 ± 1.9	4.9 ± 1.3	2.8 ± 1.8*
CPS score (0-6) mean±SD^a^	2.8 ± 2.0	4.4 ± 1.7	2.2 ± 1.8*
CPS 0-1 n (%)^b^	1027 (30.2)	75 (7.4)	952 (39.8)
CPS 2-4 n (%)^b^	1319 (38.8)	311 (30.9)	1008 (42.1)
CPS 5-6 n (%)^b^	1054 (31.0)	621 (61.7)	433 (18.1)*
Pressure ulcer n (%)^b^	345 (10.0)	191 (18.3)	154 (6.4)*
Coronary heart disease n (%)^b^	946 (27.6)	324 (31.2)	622 (25.9)**
Congestive heart failure n (%)^b^	600 (17.5)	163 (15.6)	437 (18.3)
Cerebrovascular disease n (%)^b^	836 (24.2)	345 (33.0)	491 (20.4)*
Dementia n (%)^b^	1882 (54.5)	723 (69.1)	1159 (48.2)*
Depression n (%)^b^	845 (24.6)	244 (23.4)	601 (25.1)
COPD n (%)^b^	315 (9.2)	99 (9.5)	216 (9.0)
Diabetes n (%)^b^	735 (21.4)	216 (20.7)	519 (21.7)
Parkinson’s disease n (%)^b^	254 (7.4)	98 (9.4)	156 (6.5)**
Cancer n (%)^b^	369 (10.7)	99 (9.5)	270 (11.3)
***Nutritional status***			
Weight loss n (%)^b^	312 (9.1)	146 (14.0)	166 (6.9)*
*Nutrition Therapy*			
Enteral Tube Feeding n (%)^b^	86 (2.5)	86 (8.2)	0
Parenteral Nutrition n (%)^b^	12 (0.3)	12 (1.1)	0
Texture modified diet n (%)^b^	948 (27.5)	948 (90.6)	0

^a^Student T test, ^b^chi-square test; * *p*<0,01, ** *p*<0,05

*ADL* Activities of daily living, *CPS* Cognitive Performance Scale, *COPD* Chronic obstructive pulmonary disease

At the time of the first assessment, 14.0% of the subjects with dysphagia had already registered the unintentional weight loss in the previous months compared with 6.9% of non dysphagic patients. The number of subjects who needed the assistance during meals was also significantly higher among dysphagic NHRs (90.0% vs. 51.1% respectively, *p* < 0.0001).

Dysphagia was identified as a significant risk factor for mortality. At 1-year follow up, the mortality rate in NHRs with dysphagia was statistically significantly higher than in non-dysphagic subjects (31.3% vs. 17.0%, *p* < 0.001). In the cox survival analysis NHRs with dysphagia had 58% higher risk of death within 1 year versus non dysphagic subjects (HR 1.58, 95% CI, 1.31–1.91), after adjustment for age, sex, loss of weight, pressure ulcer, congestive heart failure, cancer, COPD, disability, previous hospitalization, and nationality.

NHRs with dysphagia were mainly prescribed texture modified diets (90.6%). Less than 10% of residents with swallowing problems were treated with AN. The overall clinical conditions of subjects treated with EN and PN were particularly compromised: the prevalence of severe dementia was 86.2% compared with 59.1% in subjects with modified diet (*p* < 0.0001), and ADL disability score was higher, i.e. 5.7 against 4.8 (*p* < 0.0001).

At 1-year follow up, the prevalence of weight loss decreased in dysphagic subjects treated with AN, (from 11.0% at baseline to 9.1%) while it increased in those treated with modified diet (from 9.4% at baseline to 11.4%), but the difference was not statistically significant. Even in a logistic regression model, adjusted for statistically significant differences between the two groups—age, number of diseases, weight loss, pressure ulcers, CPS score, cerebrovascular disease, and depression—no relation between the AN and reduction of weight loss was found. Differently from the weight loss, after 12 months, the mortality was slightly higher in residents treated with AN when compared to those treated with texture modified diets (35.1% vs. 30.8%, respectively). However, the Cox survival model, after the adjustment for age, number of diseases, weight loss, pressure ulcers, CPS score, cerebrovascular disease, and depression, showed that the AN does not affect the survival (HR = 1.19 (95% CI = 0.73–1.94, p ns).

## Discussion

We retrospectively analyzed data of 3451 NHRs who participated in the SHELTER study to determine dysphagia prevalence, its association with mortality and the effect of different nutritional strategies adopted for its management on weight loss and mortality. In our study dysphagia was observed in 30.3% of NHRs, result in line with findings of previously published studies that, depending on screening and assessment tools used and on the number of participants enrolled, reported figures ranging from 9.0% to over 70% [28, 42]. As already reported in the literature, we found that dysphagia was associated with the greater occurrence of weight loss: 14.0% of subjects with dysphagia experienced weight loss in the months preceding the start of our study vs. 6.9% of non-dysphagic residents [43]. Subjects with dysphagia had a 1.58 higher 1-year risk of death compared to non-dysphagic subjects, which is again in range with findings of previously published studies [9, 32, 44–47]. Our findings on the prevalence and consequences of dysphagia confirm that it represents an issue of primary importance in NHs, which must be addressed and managed through specific protocols that provide clear indications for its diagnosis and specific management measures.

Concerning the management of dysphagia, some studies suggest that the feeding practice and assistance might be very helpful in preventing the aspiration pneumonia in subjects with dysphagia [48]. But despite the evidence supporting the effectiveness of texture modified diets in improving the nutritional status and preventing aspiration pneumonia is scant, diet modification is the most common intervention used to manage dysphagia in NHRs, as also observed in our study [34, 49–53]. On the contrary, the use of AN is a matter of intense dispute. In light of ethical considerations and considering that, according to most studies, EN and PN do not increase survival in subjects with advanced dementia, its initiation in those subjects has long been contested by most authors [54, 55]. Although other authors have shown that such criticisms are not entirely plausible, considering the low level of evidence supporting them, and that the AN does not have all the disadvantages attributed to it, this practice is actually decreasing, especially in the United States [56]. In the SHELTER cohort, texture modified diet was administered to subjects whose overall clinical conditions allowed feeding by mouth, while the AN was prescribed to only 9.4% of subjects, mostly with severe dementia. This figure is much lower than previously reported in the literature where the prevalence of AN in NHRs with dysphagia reaches up to 40.0% [32]. Nevertheless, despite the fact that the overall clinical conditions of subjects treated with AN were more compromised when compared to dysphagic residents treated with texture modified diets, we did not find that the outcomes of the two groups differed significantly. Diet modification and tube feeding did not affect differently either the loss of weight or mortality in dysphagic NHRs. These results confirm the finding of Wirth et al. who also found that the mortality rate of dysphagic residents receiving tube feeding was not significantly different from the mortality of other dysphagic residents, questioning the effectiveness of the modified diet in the prevention of weight loss in dysphagic subjects [44].

The strength of our study is that it was carried out in a large sample of European and Israeli NHRs but there are also some limitations. In the first place, dysphagia was diagnosed using only clinical observation. Observation is very important since it allows identifying signs of possible dysphagia like coughing, choking and drooling, or refusal to eat [28]. It represents indeed the most frequently used method to diagnose dysphagia in NHs. However, there are more accurate techniques and tools for dysphagia assessment, which might have identified a different and likely higher prevalence of dysphagia and provided also information on its severity [57]. EAT-10 for example is a very simple screening tool, which was found to be very effective in identifying subjects with swallowing difficulties [58–60]. Furthermore, modified diets include a large variety of diets of different textures suitable for different severity grades of swallowing problems, which we did not differentiate in this study. In addition, to evaluate the effectiveness of the modified diet in the management of the nutritional status, the calorie-protein needs, food provided and the intake should be assessed. For the assessment of nutritional status, which is an important outcome of nutrition therapy in subjects with dysphagia, no validated screening tools such as MUST or MNA were used, and only the weight loss was considered. Although numerous authors suggest the weight loss as the most relevant single indicator of nutritional status, other indicators should also be considered [61, 62].

## Conclusions

Our study, which was performed in a large sample of European and Israeli NHs, confirmed that dysphagia is a common problem in that setting and that it represents an important risk factor for mortality. The main interventions adopted for dysphagia management are the modification of diet texture and AN but clinical outcomes—weight loss and mortality – of the subjects from the two intervention groups do not differ significantly. Further studies are needed to clarify the correct indications for diet consistency changes and AN in this vulnerable population.

## Data Availability

The datasets generated and/or analyzed during this current study are not publicly available due to ongoing use of data set but are available from the corresponding author on reasonable request.

## Abbreviations

NH: Nursing home
NHRs: Nursing home residents
AN: Artificial nutrition
EN: Enteral nutrition
PN: Parenteral nutrition
LTCF: Long term care facility
MMSE: Mini mental state examination
ADL: Activities of daily living MMSE
CPS: Cognitive Performance Scale
COPD: Chronic obstructive pulmonary disease
